# Robust Optimization Model and Algorithm for Railway Freight Center Location Problem in Uncertain Environment

**DOI:** 10.1155/2014/607159

**Published:** 2014-11-04

**Authors:** Xing-cai Liu, Shi-wei He, Rui Song, Yang Sun, Hao-dong Li

**Affiliations:** ^1^School of Traffic and Transportation, Beijing Jiaotong University, Beijing 100044, China; ^2^Integrated Transport Research Center, China Academy of Transportation Sciences, Beijing 100029, China

## Abstract

Railway freight center location problem is an important issue in railway freight transport programming. This paper focuses on the railway freight center location problem in uncertain environment. Seeing that the expected value model ignores the negative influence of disadvantageous scenarios, a robust optimization model was proposed. The robust optimization model takes expected cost and deviation value of the scenarios as the objective. A cloud adaptive clonal selection algorithm (C-ACSA) was presented. It combines adaptive clonal selection algorithm with Cloud Model which can improve the convergence rate. Design of the code and progress of the algorithm were proposed. Result of the example demonstrates the model and algorithm are effective. Compared with the expected value cases, the amount of disadvantageous scenarios in robust model reduces from 163 to 21, which prove the result of robust model is more reliable.

## 1. Introduction

The location problem is one of the most studied issues in combinatorial optimization, which is widely applied in communication industry, transportation, and logistics industry. In China, railway freight transport center specifies the railway freight station, which is equipped with various kinds of facilities. Recently, many railway freight transport centers have been constructed for the purpose of centralized and express transportation. The railway freight transport center location problem is very crucial for the construction of railway freight transport center, which is costly and influential.

Many models have been set up to study this problem such as covering model, *p*-median model, and *p*-center model [1–4]. Recently, Racunica and Wynter [5] used two variable-reduction heuristics to solve the hub location problem in intermodal transport hub-and-spoke networks. Jesús and Paula [6] added a coverage constraint to the *p*-median model and applied three different algorithms to solve it.

Most of the research in literature studied this problem in certain environment. However, many elements in the location problem are fluctuant, especially the transport demand. So the reliability problem of location result was proposed [7]. Chance constraint model is a way to solve the uncertain problem which uses expected value, chance measure and realization probability to investigate the situation. Chance constraint model needs the distribution function of the uncertain element which is difficult to measure. Meanwhile, the distribution function cannot include all situations. The service quality will be affected by the negative scenarios, whose demand is beyond the distribution function. However, robust optimization model can largely avoid this dilemma. Both expected objective value and deviation between actual objective value and expected value are considered [8, 9]. The result can decrease the occurrence of negative scenarios. Robust optimization has been used in network plan [10], routing optimization [11], scheduling problem [12], and so forth. In China, Wang and He [13] used chance constraint model to solve railway logistic center location problem. Sun et al. [14] applied the robust optimization on the feeder bus network timetable schedule problem.

The main purpose of this paper is to provide robust optimization model of railway freight transport center location problem and a method to solve it. The location optimization model considers service coverage constraint. The adaptive clonal selection algorithm (ACSA) is combined with the Cloud Model (CM) called cloud adaptive clonal selection algorithm (C-ACSA) to solve the model. The outline of this paper is as follows: Section 2 introduces the robust optimization model of freight center location problem. In Section 3, a new algorithm is proposed. Finally, a numerical example is given to illustrate the application of the model and algorithm.

## 2. Robust Optimization Model of Railway Freight Transport Center Location Problem

(*1) Decision Variables.* Scenario specifies the realization of stochastic demand. And transport demand of the scenario is known. The objective of robust model is to find the location of railway freight transport centers and the assignment between centers and shippers in all scenarios. The location decision and assignment are treated as decision variables. Those are as follows.

 
*x*_*ij*_^*k*^ equals 1 if shipper *i* is assigned to center *j* in scenario *k*. Otherwise, it equals 0.
 
*y*_*j*_^*k*^ equals 1 if a railway freight transport center is located at candidate center *j* in scenario *k*. Otherwise, it equals 0.

(*2) Objective Function*

(*a) Objective Function of Deterministic Model.* Cost of location problem in scenario *k* includes two parts: the first is construction cost of railway freight transport centers; the second is transport cost between shippers and the centers. The objective function of scenario *k* is as follows:

(1) $$\begin{array}{c} z_{k}=\mu_{1}c\underset{i\in I}{\sum }\;\underset{j\in J}{\sum }h_{i}^{k}d_{ij}x_{ij}^{k}+\mu_{2}\underset{j\in J}{\sum }C_{j}y_{j}^{k}, \end{array}$$

where *c* is unit transport cost of transport demand from shipper to railway freight transport center. *μ*_1_ and *μ*_2_ are weight of transport cost and construction cost in objective function. They are defined in advance. And *μ*_1_ + *μ*_2_ = 1, *μ*_1_ ≥ 0, *μ*_2_ ≥ 0. *h*_*i*_^*k*^ is transport demand of shipper *i* in scenario *k*. *d*_*ij*_ is distance between shipper *i* and railway freight transport center*j*. *C*_*j*_ is fixed cost to construct a center at candidate center *j*. *I* is set of shippers, *i* ∈ *I*. *J* is set of candidate centers, *j* ∈ *J*. 

(*b) Objective Function of Robust Optimization Model.* To set up robust optimization model, expected optimization model should be set at first. Define *δ*(*k*) as the probability of scenario *k*, which means the realization probability of the scenario. *K* is set of scenarios. Expected value of optimization model is as follows:

(2) $$\begin{array}{c} E\left(z\right)=\mu_{1}c\underset{k\in K}{\sum }\;\underset{i\in I}{\sum }\;\underset{j\in J}{\sum }h_{i}^{k}d_{ij}x_{ij}^{k}\delta \left(k\right)+\mu_{2}\underset{k\in K}{\sum }\;\underset{j\in J}{\sum }C_{j}y_{j}^{k}\delta \left(k\right). \end{array}$$

The robust optimization model further measures the deviation between expected and actual objective values. If actual objective value *z*_*k*_ is worse than the expected value *E*(*z*), scenario *k* will influence the optimized result. So only the *z*_*k*_ which is worse than *E*(*z*) is considered in the deviation Δ:

(3) $$\begin{array}{c} \Delta =\underset{k\in K}{\sum }\max\left(0,z_{k}-E\left(z\right)\right)\delta \left(k\right). \end{array}$$

Objective function of robust optimization model can be presented as follows:

(4) $$\begin{array}{c} Z=E\left(z\right)+\kappa \Delta , \end{array}$$

where *κ* is weight of the deviation value in the objective.

(*3) Constraints*

(a) Each shipper must be assigned to one freight transport center in scenario *k*:

(5) $$\begin{array}{c} \underset{j\in J}{\sum }x_{ij}^{k}=1\;\forall i\in I,\;\;k\in K. \end{array}$$

(b) Candidate center *j* cannot serve any shipper, if *j* is not chosen as a freight transport center:

(6) $$\begin{array}{c} x_{ij}^{k}\leq y_{j}^{k}\;\forall i\in I,\;\;j\in J,\;\;k\in K. \end{array}$$

(c) The total number of chosen freight transport center should be constrained:

(7) $$\begin{array}{c} \underset{j\in J}{\sum }y_{j}^{k}\leq p\;\forall k\in K, \end{array}$$

where *p* is maximum number of chosen freight transport center, which is preestablished.

(d) The sum of distance which is greater than coverage distance *DC* at a freight transport center should not exceed *ε*. Both *DC* and *ε* are prespecified:

(8) $$\begin{array}{c} \underset{i\in I}{\sum }l_{ij}x_{ij}^{k}\leq \varepsilon \;\forall j\in J,\;\;k\in K. \end{array}$$

The coefficient *l*_*ij*_ is defined as follows:

(9) $$\begin{array}{c} l_{ij}=\left\{\begin{array}{cc} d_{ij} & d_{ij}>DC\\ 0 & \text{otherwise}. \end{array}\right. \end{array}$$

(f) The transport demand serviced by freight transport center *j* cannot exceed its capacity Cap_*j*_:

(10) $$\begin{array}{c} \underset{i\in I}{\sum }h_{i}^{k}x_{ij}^{k}\leq \text{Ca}{\text{p}}_{j}\;\forall j\in J,\;\;k\in K. \end{array}$$

(*4) The Robust Optimization Mathematical Model.* The robust optimization model of freight transport center location problem can be stated as follows:

(M-I)

(11) Min⁡ Z=μ1c∑k∈K ∑i∈I ∑j∈Jhikdijxijδk+μ2∑k∈K ∑j∈JCjyjkδk+κ∑k∈Kmax⁡0,zk−E(z)δ(k)s.t. formulas  (5)–(8),(10)xijk∈0,1 ∀i∈I,  j∈J,  k∈Kyjk∈0,1 ∀j∈J,  k∈K.

## 3. Solution Algorithm

ACSA [15–17] has clone, mutation, and selection operations. It is shown to be an evolutionary strategy which has high convergence rate and diversified antibodies. CM is proposed by Li and Du [18], which is used to convert the qualitative data into quantitative data. It is widely applied in many fields such as evolutionary algorithm, intelligent control, and fuzzy evaluation. CM has the character of randomness and stable tendentiousness. It can be used to control the direction of search and improve the convergence rate, according to the affinity of the antibody. The ACSA is combined with CM into a new heuristics, called C-ACSA method. This method has variety species group and can balance the local search and global search.

### 3.1. The Detail Techniques of ACSA

**Figure 1:**
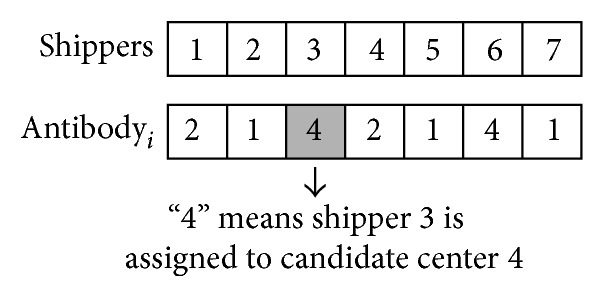
The design of antibody for the optimization model.

**Figure 2:**
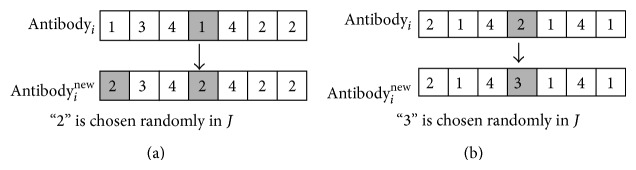
The mutation operation of model M-I.

(*1) Affinity Measure.* Affinity of the algorithm is the objective of model, the smaller the better. In order to extend the search space, the algorithm accepts solutions which fail to satisfy the constraints. However, penalty coefficient will be added to the affinity measure.

(*2) The Design of Antibody.* The length of antibody equals the amount of shippers in *I*. The antibody codes are in *J*, and the amount should not exceed the maximum number *p*. To better understand the design of antibody, a simple example consisting of seven shippers and four candidate freight transport centers is proposed. *p* equals three (see Figure 1). Candidate center 3 is not included in the antibody, which means candidate center 3 is not chosen as a transport center.

(*3) Mutation Operation.* The mutation operation is shown in Figure 2. *p* equals four. If the amount of chosen candidate centers reaches maximum, randomly choose a code *e*. Change both *e* and the codes whose values are the same as *e* (see Figure 2(a)). Else randomly choose a code *e* and change its value (see Figure 2(b)).

### 3.2. Cloud Model

**Figure 3:**
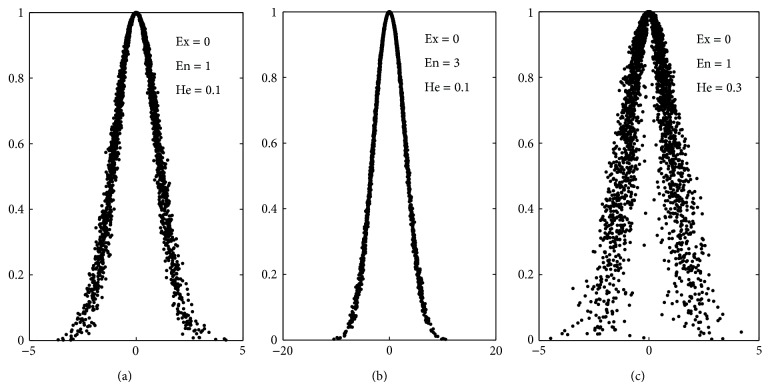
Three examples of the Normal Cloud.

(*1) Cloud Model.* CM is used to transform the qualitative data into quantitative data. A Cloud Drop is a realization of the qualitative concept; the distribution of Cloud Drops is called Cloud. Three numerical characteristics are used to describe the Cloud; those are expected value *Ex*, entropy *En*, and hyper entropy *He*. The typical CMs are Normal Cloud, Trapezoid Cloud, and Triangle Cloud. If distribution function of Cloud follows the normal distribution, the CM is called Normal Cloud. Three Normal Clouds with different characteristics are shown in Figure 3. Compared the three Clouds, it can be found that the bigger the characteristics are, the more divergent the Cloud will be.

The characteristics of Normal Cloud can be got by the following operations:

(12) $$\begin{array}{c} Ex=\bar{f},\\ En=\frac{\left(\bar{f}-f_{\min}\right)}{c_{1}},\\ He=\frac{En}{c_{2}}, \end{array}$$

where *c*_1_ and *c*_2_ are control coefficients. $\bar{f}$ is the average value of affinities in the group. *f*_*i*_ is affinity of the antibody. *f*_min⁡_ is the minimum affinity of the antibody.

(*2) Cloud Generator. *Cloud Generator (CG) is the algorithm of CM. The inputs of the generator are the three numerical characteristics. The outputs are Cloud Drops. CG can realize the mapping from qualitative data to quantitative data. There are many CGs such as Forward Cloud Generator, Backward Cloud Generator, X Condition Cloud Generator, and Y Condition Cloud Generator. The Forward Cloud Generator is used to generate Cloud Drops based on the samples which are in set (*Ex*, *En*, and *He*). The Cloud Drops can be got by the following formulas:

(13) $$\begin{array}{c} {En}^{\prime }=\text{NORM}\left(En,{He}^{2}\right),\\ Q_{\text{cloud}}=e^{-{\left(f_{i}-Ex\right)}^{2}/2{En}^{\prime 2}}. \end{array}$$

*Q*_cloud_ is a Cloud Drop which means the uncertainty degree of the inputs, *Q*_cloud_ ∈ (0,1).

## 4. Progress of the Algorithm

C-ACSA combines the advantages of CM and ACSA. The design of algorithm parameters considers the randomness and stableness of Cloud Drop. The mutation and clone rates are big at the initial stage of the algorithm; so antibody with low affinity has the chance to clone and evolve, which helps to extend the search space. At the late stage of the algorithm, the mutation and clone rates are small; so antibody with big affinity is protected and global convergence rate is accelerated.

Based on the aforementioned detailed analysis, C-ACSA approach can be designed as the following procedure.

**Step 1 .**

Initialize the group of antibody. Generate *N* antibodies and constitute the species group *P*.

**Step 2 .**

Count the affinities and sort antibodies according to their affinities in an ascending order.

**Step 3 .**

Clone each antibody in *P* and then get a new species group *C*. The number of clone is *n*_*i*_ = ⌊*w*_max⁡_(1 − (*i* − 1)/*N*)⌋ and *n*_*i*_ ≥ *w*_min⁡_, where *i* is the sequence of antibody after sorting. *w*_max⁡_ is the maximum clone number, *w*_min⁡_ is the minimum clone number, and ⌊⌋ means rounding.

**Step 4 .**

Use mutation operation to update each antibody in *C*. And get the new species group *C*′. The mutation rate is inversely proportional to evolution generation *φ*_*l*_^*i*^ = ⌊*Q*_cloud_(1 − *l*/*L*)⌋, where *l* is the current generation and *L* is the maximum generation.

**Step 5 .**

Choose the first *d*_*l*_ antibodies in *C*′ and replace the worst *d*_*l*_ antibodies in *P* by them, $d_{l}=\left\lfloor \left(\overset{-}{f}-f_{\min}\right)\left(D/\bar{f}\right)\right\rfloor$, where *D* is the coefficient, $\overset{-}{f}$ is the average value of affinities in *C*′, and *f*_min⁡_ is the minimum value of affinities in *C*′.

**Step 6 .**

If current status does not meet the terminal condition (the maximum computing times), go to Step 2. Otherwise, go to Step 7.

**Step 7 .**

Output the best solution, that is, the optimal location of freight centers.

## 5. Numerical Experiment

In order to show the efficiency and effectiveness of the proposed model and approach, this section applies the model and C-ACSA to optimize the location of centers. In the programming area, there are 23 shippers and 7 candidate freight transport centers; distances between shippers and railway freight transport centers are shown in Table 1. The distances satisfy the triangle inequality. The distributions of transport demand are shown in Table 2, and the distributions are homogeneous distribution. The parameters of the optimal model are *c* = 0.1 ((million CNY)/(km^−1^ *·*Mt^−1^)). *μ*_1_ = 0.6, *μ*_2_ = 0.4, *p* = 4, *ε* = 15, *DC* = 12, Cap_*j*_ = 40 (Mt), and *C*_*j*_ = 100 (million CNY).

The parameters of the C-ACSA are *N* = 20, *w*_max⁡_ = 8, *w*_min⁡_ = 2, *L* = 100, *D* = 10, *c*_1_ = 60, and *c*_2_ = 10. Using *C*# to solve the experiment. 300 scenarios were simulated stochastically and the model was solved under three weights of *κ* which were 0, 10, and 20. When *κ* is 0, the robust model is expected optimization model. The result of location problem is shown in Table 3. The computing time is around 2 s. Also, ILOG Cplex program is devised. The optimization result is the same, but Cplex cannot provide the amount of the disadvantage scenarios.

As is shown in Table 3, the location result of robust optimization model chose one more freight transport center than the expected optimization model, which means it needs more centers to make up the influence of stochastic demand. The number of disadvantageous scenarios in expected value model is the maximum; there are 163 disadvantageous scenarios in total 300 imitation scenarios. Compare the results with different *κ* values, when *κ* increases the expected value of model increases, while the deviation value and the disadvantageous scenarios decrease. So the introduction of robust model improves transport capacity of the system, which makes the location result more reliable and more applicable. Furthermore, the increase of *κ* will decrease the deviation value, which needs more investment and causes the expected value to increase. In practice, the planners need to decide the index *κ* and balance the weight between expected value and deviation value.

## 6. Conclusion

A robust optimization model is proposed to mitigate the influence of disadvantageous scenarios which is caused by the stochasticity of the transport demand. The robust model is based on the deterministic model and expected optimization model. A new heuristic algorithm is proposed which combines CM with ACSA. The numerical example is implemented on a network. Computational results demonstrate the model and algorithm are available. And the robust model can help to improve the reliability of location decision. While there are some fluctuations such as transport cost, constructing cost that are not considered in the model. These aspects can be considered in the future research.

## Figures and Tables

**Table 1:** Distances between shippers and candidate centers (km).

Center	Shipper																						
	1	2	3	4	5	6	7	8	9	10	11	12	13	14	15	16	17	18	19	20	21	22	23
1	40	3	5	4	3	6	4	9	15	27	20	27	21	25	25	29	30	35	38	42	20	13	24
2	120	11	13	12	11	14	4	1	7	19	12	19	13	17	17	21	22	27	30	34	12	5	16
3	250	23	26	25	23	27	17	12	6	6	3	7	16	20	20	24	10	15	15	19	18	14	3
4	310	20	32	31	29	33	22	17	15	14	8	10	19	23	23	27	5	10	10	14	18	16	11
5	290	28	30	29	28	31	22	15	13	12	6	8	17	21	21	25	7	12	12	16	16	14	9
6	120	11	13	12	8	14	15	10	16	28	21	13	12	16	16	20	25	30	30	34	8	7	25
7	160	15	25	24	21	23	22	17	25	35	23	4	6	10	11	14	28	33	34	39	5	9	32

**Table 2:** The distribution of transport demand (Mt).

Shipper	1	2	3	4	5	6	7	8	9	10	11	12
Demand	[3, 8]	[0.5, 2]	[2, 5]	[1, 4.5]	[4, 9]	[3, 6]	[3, 8]	[2, 7]	[1, 5]	[4, 7]	[3, 7]	[1, 5]

Shipper	13	14	15	16	17	18	19	20	21	22	23	
Demand	[1, 4.5]	[5, 10]	[0.5, 4]	[2, 7]	[3, 9]	[0.5, 5]	[2, 6]	[1, 5]	[2, 6]	[1, 7]	[2.5, 5]	

**Table 3:** The location result of robust model with different *κ*.

*κ*	Result	Assignment of the shippers	The expected value	The expected value of deviation	The amount of the disadvantageous scenarios
0	1	1, 2, 3, 4, 5, 6, 7, 8	3186.9	587.97	163
	3	9, 10, 11, 17, 18, 19, 20, 23			
	7	12, 13, 14, 15, 16, 21, 22			

10	1	1, 2, 3, 4, 5, 6, 7, 8	4094.9	9.88	21
	3	9, 10, 11, 23			
	4	7, 18, 19, 16			
	7	12, 13, 14, 15, 16, 21, 22			

20	1	1, 2, 3, 4, 5, 6, 7, 8	4094.9	9.88	21
	3	9, 10, 11, 23			
	4	7, 18, 19, 16			
	7	12, 13, 14, 15, 16, 21, 22			
